# Does customised care improve satisfaction and positively enable parents in sustained home visiting for mothers and children experiencing adversity?

**DOI:** 10.1186/s12913-022-08759-9

**Published:** 2022-11-16

**Authors:** Kie Kanda, Stacy Blythe, Rebekah Grace, Emma Elcombe, Lynn Kemp

**Affiliations:** 1 School of Nursing and Midwifery, Translational Research and Social Innovation Group, Western Sydney University, Ingham Institute for Applied Medical Research, Level 3, 1 Campbell Street, Liverpool, NSW 2170 Australia; 2 Transforming Early Education and Child Health, Translational Health Research Institute, Western Sydney University, Campbelltown, NSW 2560 Australia

**Keywords:** Customised care, Satisfaction, Enablement, Maternal and child care, Sustained nurse home visiting, Quality of care

## Abstract

**Background:**

The Maternal Early Childhood Sustained Home-visiting program (MECSH) is a structured nurse-delivered program designed to address health inequities experienced by families experiencing significant adversity. There is strong evidence for the effectiveness of this program, but limited research exploring the practice and process elements that are core to positive parent outcomes. This study aimed to examine the relationship between customised care related to the mother’s risk factors and parent satisfaction and enablement in the delivery of a MECSH-based program.

**Methods:**

A cross-sectional study design was used. Program delivery data collected as part of a large randomised controlled trial of a MECSH-based sustained nurse home visiting program in Australia (right@home) were analysed. This study used the data collected from the intervention arm in the trial (*n* = 352 women). Parent satisfaction was measured at child age 24 months using the modified short-form Patient Satisfaction Questionnaire. Parent enablement was measured at child age 24 months by a modified Parent Enablement Index. Customised care was defined as appropriate provision of care content in response to four maternal risks: smoking, mental health, domestic violence and alcohol and drugs. Logistic analysis was performed to assess the impact of customised care on parent satisfaction and enablement while adjusting for covariates such as sociodemographic factors. A significance level of 95% was applied for analysis.

**Results:**

Our results indicated high levels of satisfaction with the care provided and positive enablement. There were several sociodemographic factors associated with satisfaction and enablement, such as language spoken at home and employment experience. The mothers who received customised care in response to mental health risk and domestic violence had significantly greater satisfaction with the care provided and experienced an increase in enablement compared to those who did not receive such care.

**Conclusion:**

This study contributes to the existing body of empirical research that examines the relationship between care processes and client outcomes in the delivery of home visiting services. It is essential for the sustained nurse home visiting service model to be flexible enough to cater for variations according to family circumstances and needs while maintaining a core of evidence-based practice.

## Background

Sustained nurse home visiting is a service delivery mechanism that has been used to provide prevention and intervention services in which mothers, caregivers and children receive structured support for health and well-being within their home environment over an extended period of months or years [1–4]. Based on an increasing body of research evidence on the effectiveness of sustained home visiting programs, they are considered an impactful strategy for addressing the multiple and complex needs of families who are experiencing adversity [3, 5–8]. However, despite the strength of the evidence base, we still do not fully understand the practice and process elements that are core to positive parent outcomes.

The Maternal Early Childhood Sustained Home-visiting (MECSH) program was developed and trialled in Australia and is being delivered across the world. It is a structured program designed to address health inequities by providing intensive professional support for maternal and child health and well-being and child development for families who are at significant risk of poor health and developmental outcomes [4, 9]. The services are delivered by university-trained registered nurses with additional (usually postgraduate) training in child and family health nursing. The nurses are also provided additional online and face-to-face training in the MECSH program as well as reflective practice supervision. The MECSH program commences in pregnancy and continues until the child is two years old. Previous research evidence has shown MECSH-based programs to be effective, reporting significant improvements in maternal confidence in care, knowledge and experience, positive child health and development outcomes, and creating positive home environments to support healthy child development [4, 10–12].

In order to provide meaningful and individualised care, the MECSH program encourages and supports home visiting nurses to purposefully adapt and customise the care provided for mothers and families, without compromising core program components and fidelity [13]. Care modification, however, can happen in different ways, and can be both deliberately planned or spontaneous, and with both positive and negative outcomes. If a variation in the care delivery is unwarranted, it may leave families at risk of not receiving appropriate care [14]. On the other hand, when a variation is well-planned and it is warranted to customise or tailor the program to meet the client’s unique needs and preferences [15], the variation can be considered ‘purposeful’, and expected to enhance outcomes. The theoretical concept of ‘precision home visiting’ is underpinned by the idea that there is a need to achieve an appropriate balance between program compliance and purposeful variation. Precision home visiting is home visiting that differentiates what works, for whom, and in what contexts to achieve specific outcomes [16, 17]. It focuses on the components of home visiting services that are most likely to be effective in light of mothers’ and families’ characteristics and social and cultural context [18]. A previous study [19] found that home visiting nurses were actively engaged in ensuring the care they provided had high fidelity to the program in terms of scheduled content, but also engaged in purposeful variation based on maternal risk. The mothers who were at high risk of smoking, mental health issues, domestic violence, and substance abuse were more likely to receive specific care content related to these risks than mothers who did not have increased risk on these factors. The study suggested a need for more research on the relationship between purposeful variation and the critical outcomes of care.

A study by O’Brien and colleagues [20] noted that purposeful variation was associated with higher program retention: nurses in high retention rate sites adapted the program to their clients’ needs, were less directive, and more collaborative. Furthermore, the flexible style of practice may be more appreciated by higher-risk mothers, who are likely to experience uncertain situations [20]. Two other key outcome measures of healthcare quality are client satisfaction and enablement [21, 22]. Client satisfaction is defined as a health care recipient’s satisfaction with salient aspects of the context, process, and service experience [23]. It is considered a nursing-sensitive client outcome, which is significantly impacted by nursing interventions [24]. Client enablement is defined as a client’s ability to understand and manage their health and life [25]. It arguably goes beyond measures of satisfaction in that it requires clients to make judgements about their own intervention outcomes rather than simply evaluating aspects of the practitioner’s performance [25]. These critical outcomes provide insightful measures of client perceptions of the care processes and their sense of empowerment resulting from increased knowledge, understanding and improved capacity for managing health and well-being [26].

Several previous studies have demonstrated that home visiting interventions have higher levels of client satisfaction with care than facility-based services [11, 12, 27–29]. In addition, qualitative research evidence supports that parent who received home visiting services valued the relationships they developed with nurses and were satisfied that the care processes were respectful, empowering, and provided emotional support [30–33]. There is limited research focused on parent enablement as the result of home visiting care services. However, Kemp and colleagues [34] reported in their trial of the MECSH program that intervention women indicated feeling significantly more enabled to cope with and understand their baby and care for themselves and their baby than the comparison group who received usual care services. This was associated with the provision of psychosocial support and continuity of home visiting nurses during the pre and postnatal periods. This is consistent with another study demonstrating that positive professional communication and continuity of care are associated with higher levels of client enablement [35]. Previous studies in the clinical setting suggest that enablement is correlated with process factors such as degree of client-centredness, a positive approach [36], and practitioner empathy [37].

In summary, previous research on the importance of customised care generally focuses on the clinical setting. There is little evidence on the impact of customised care on client’s key outcomes such as satisfaction and enablement as it relates to home visiting. Therefore, this paper seeks to contribute to the small existing body of research that empirically examines the impact of care processes and practices in the delivery of maternal and early childhood home visiting services on parent satisfaction and enablement.

### Study aim

The major aim of this study was to examine the relationships between customised care in response to mothers’ risk factors, and parent satisfaction and enablement in the delivery of a MECSH-based program. The study was guided by the following research questions:

i) How satisfied are the clients of the MECSH-based program with the care provided, and to what extent do these clients report parent enablement?
ii) What is the relationship between sociodemographic characteristics and parent satisfaction and enablement?
iii) What is the relationship between customised care in response to the mothers’ risk factors and parent satisfaction?
iv) What is the relationship between customised care in response to the mothers’ risk factors and parent enablement?

## Methods

### Study design

A cross-sectional study design was used. This study drew on data collected from the intervention arm of a randomised controlled trial (RCT) of a MECSH-based program, namely right@home. This sustained nurse home visiting program was trialled in seven localities in the Australian states of Victoria and Tasmania, where nurses worked with families from pregnancy to child age two years [38].

### Participants

Eligibility criteria for the right@home trial included pregnant women attending the antenatal clinics in Victoria and Tasmania from May 2013 to August 2014, who were less than 37 weeks gestation, had sufficient English proficiency to verbally answer interview questions, resided within the study travel boundaries, and reported two or more of ten sociodemographic risk factors for adverse parent and/or child outcomes in the risk factor screening. These risk factors included: young pregnancy (age < 23 years); not living with another adult; no support in pregnancy; smoking; poor/fair/good health; long-term illness; anxious mood; not completed secondary level education; no income; and never worked [38–40]. The right@home trial recruited 722 pregnant women. Randomization was stratified by geographic region and parity. The intervention arm of the trial recruited 363 families, and 352 women commenced the intervention. The comparison group received usual care [38]. This study used the data collected from the intervention arm only as it was focused on the intervention program content, and therefore not relevant to the comparison group who did not receive the program.

### Measures

#### Participants sociodemographic characteristics and risk factors

Women’s data collected at program commencement, including sociodemographic characteristics and risk factor screening data, and children’s dates of birth were extracted from the trial enrolment data.

#### Visit content provided for mothers and families

As part of the study, visit content was recorded by the nurses at the completion of each visit [11, 41]. The nurses completed an online checklist designed explicitly for use in program quality monitoring. It included the unique client identifier, visit date, and the activities undertaken. The electronic checklist was located on the nurse’s mobile device (tablet) with a simple touch entry. There were 48 content items in the antenatal checklist and 56 content items in the postnatal checklist [11, 41].

#### Customised care measure

The existence of four risk factors; mental health issues, domestic violence, smoking, and alcohol and drugs, were identified by using the data collected at the commencement of the program and information disclosed by families and recorded in client notes by the intervention nurses during the course of home visits. Mental health risk was screened using Depression Anxiety Stress Scales (DASS) [42] (cut-off for Anxiety ≥ 10; Depression ≥ 14; Stress ≥ 19) at commencement of program and client disclosure of a mental health diagnosis. The other three risks were identified at the baseline interview via questions regarding each risk. For example, mothers were asked if they had experienced a drinking problem in the previous year. Client disclosure of self-reported risk related to domestic violence, smoking, and drugs and alcohol during the home visits were also considered indicative of risk. Nurse records were collected through a nurse-reported record audit and collated with risk information collected by the researchers.

**Fig. 1:**
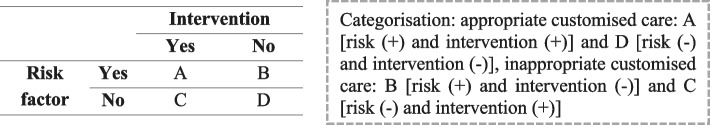
Categorisation of appropriate and inappropriate customised care

The provision of appropriately customised care was based on these risk factors and the care content data. We considered that the mother received appropriately customised care based on the presence or absence of risk factors, if at least one episode of care content related to her risk factors was or was not provided to her for the entire period from the commencement till the completion of the program intervention at child age 24 months. Content data was aggregated over multiple visits. Mothers who received appropriately customised care based on their risk factors, were categorised into two groups (See Fig. 1): appropriate customised care group is the combination of A and D, and inappropriate customised care group is the combination of B and C.

As reported in the previous research, of the total participants who completed the intervention (*n* = 304), the prevalence of the four risks in the study were: smoking *n* = 98 (32%), mental health *n* = 203 (67%), domestic violence *n* = 88 (29%), and drugs and alcohol *n* = 163 (54%) (See the reference [19] for details).

#### Parent satisfaction

The level of parent satisfaction was measured at child age 24 months using the modified short-form Parent Satisfaction Questionnaire (PSQ-18) [27], which was based on the short-form Patient Satisfaction Questionnaire (PSQ) [43]. Ten items measured parent satisfaction with the services provided by the nurse as this relates to communication, interpersonal manner, time spent, accessibility, and general satisfaction. Each domain is rated on a 5-point scale of ‘strongly disagree’ (1 score) to ‘strongly agree’ (5 score). Five questions are reverse scored. Scores were summed to give total scores which range from 10 to 50 and subscales scores which range from 2 to 10. A total score less than 20 was considered at the level of concern. A previous home visiting study demonstrated PSQ to have high interdependence (Cronbach alpha coefficient 0.84) [27].

#### Parent enablement

Parent enablement was measured at child age 24 months using a modified Parent Enablement Index (PEI) [25, 38, 44]. It included six items assessing the degree of mother’s feeling of enablement; whether, as a result of the program, mothers felt better able to cope with life, understand her baby, cope with her baby, keep herself healthy, and felt confident about her health, and able to help herself. Each question is rated on a 3-point scale which is ‘much better’ (2 score), ‘better’ (1 score), and ‘same or less’ (0 score). Scores were summed to give a total enablement score with the range from 0 to 12. A total score greater than 4 was considered that the mother felt more enabled by the service.

### Analytic strategy

Data were analysed using R (version 4.0.5) and the R packages ‘ordinal’ and ‘moments’. Data were summarised using descriptive statistics, including frequencies, means, standard deviations and percentages as appropriate. The PSQ and PEI scores were shown to have non-normal data distributions (PEI: skewness = 0.60, *p* < 0.001, kurtosis = 2.18, *p* < 0.001, PSQ: Skewness = -1.21, *p* < 0.001, kurtosis = 5.27, *p* < 0.001). As such, the associations between these primary outcome measures and the demographic and customised care provision were assessed using the Mann–Whitney U test of independence [45].

Ordinal logistic regression was performed to assess the impact of customised care on PSQ and PEI while adjusting for the original sample stratification factors (geographic area and parity) and the sociodemographic factors shown to be of relevance. Models were fitted using restricted maximum likelihood (REML). Geographic region was fitted as a random effect, while parity, a binary variable, was included as a fixed factor.

## Results

### Level of parent satisfaction and enablement

Of the 352 women who commenced the intervention, 304 (86%) women completed the right@home program when the child reached two years of age. Of 304 women, 261 (86%) and 265 (87%) women completed the PSQ and PEI questionnaire, respectively, at child age two years. As shown in Table 1, overall results indicated high levels of satisfaction with the care provided, with a mean PSQ score of 44.77 (SD = 5.43). Only 1 (0.4%) response was at the level of concern (score less than 20). Among five subscales of PSQ, Communication had the highest mean score of 9.08 (SD = 1.12), and Accessibility and Convenience were the lowest with a mean of 8.69 (SD = 1.36). The mean PEI score was 4.31 (SD = 4.00). Altogether 72 of 265 participants (27%) had the floor score (0 points) and 29 (11%) the ceiling score (12 points). A total of 114 participants (43%) rated that they felt more enabled by the service (total score greater than 4).

**Table 1:** Patient Satisfaction and Enablement scores at child age 2 years

Total PSQ score	Min	Median	Max	IQR	Mean	SD
	10	46	50	10	44.77	5.43
***PSQ – subscales***						
Communication	2	10	10	2	9.08	1.12
General satisfaction	2	9	10	2	8.96	1.31
Interpersonal manner	2	9	10	2	9.04	1.06
Time spent	2	10	10	2	8.98	1.36
Accessibility and Convenience	2	9	10	2	8.69	1.36
**Total PEI score**	0	3	12	7	4.31	4.00

### Association between sociodemographic characteristics and parent satisfaction and enablement

Table 2 shows the total PSQ and PEI scores by sociodemographic characteristics. Mothers who spoke a language other than English at home, and those who had never had a job before, had significantly lower PSQ scores than those who spoke English at home and had experience with paid employment. Conversely, mothers speaking a language other than English at home had significantly higher PEI scores than those speaking English at home.

**Table 2:** Patient satisfaction and Enablement by sociodemographic characteristics

**Characteristics**	**PSQ (*n* = 261)**						**PEI (*n* = 265)**					
	n	mean	SD	median	IQR	P*	n	mean	SD	median	IQR	P*
***Age***												
Young pregnancy (< 23 years)^a^	60	44.9	5.8	47.0	10.0	> 0.1	60	4.3	4.4	3.0	7.25	> 0.1
More than 23 years old	201	44.7	5.3	46.0	9.0		205	4.3	3.9	3.0	7.0	
***Language spoken at home***												
English	240	45.0	5.4	47.0	9.0	**0.005**	244	4.2	4.1	3.0	7.0	**0.006**
Other than English	21	42.1	4.8	40.0	6.0		21	6.1	2.7	6.0	3.0	
***Education***												
Completed secondary education	125	44.9	5.2	47.0	9.0	> 0.1	127	4.3	3.9	3.0	7.0	> 0.1
Less than secondary education	136	44.6	5.6	46.0	10.0		138	4.3	4.1	3.0	7.0	
***Parity***												
First child	102	44.8	5.4	46.0	9.75	> 0.1	103	4.7	4.1	4.0	7.0	> 0.1
Not first child	159	44.7	5.5	47.0	9.5		162	4.0	3.9	3.0	6.0	
***Living with partner***												
Yes	185	44.8	6.3	46.0	10.0	> 0.1	189	4.3	3.9	3.0	7.0	> 0.1
No	76	44.6	5.1	46.5	9.0		76	4.3	4.2	3.0	6.25	
***Paid work***												
No person in HH^b^ has paid work	80	44.8	5.5	47.0	10.0	> 0.1	80	4.8	4.3	5.0	7.25	> 0.1
Someone in HH^b^ has paid work	181	44.8	5.4	46.0	9.0		185	4.1	3.8	3.0	6.0	
***Employment experience***												
Never had a job before	44	43.0	5.1	41.0	8.0	**0.003**	44	3.8	3.6	3.5	6.0	> 0.1
Had a job before	217	45.1	5.4	47.0	9.0		221	4.4	4.1	3.0	7.0	

^a^the age cut-off (< 23 years) was used by the enrolment screening in the program

^b^*HH* Household, *IQR* Interquartile range, *SD* Standard Deviation

^*^Mann–Whitney U test performed

### Relationship between appropriate customised care and parent satisfaction and enablement

Table 3 shows the PSQ and PEI scores by the provision of appropriate customised care in response to risk factors. The mothers who received appropriate customised care related to mental health risk had significantly greater satisfaction with the care provided than those who did not receive appropriate customised care. However, the mothers who received appropriate customised care in response to smoking risk reported significantly lower enablement than those who did not have appropriate customised care. There was a tendency for customised care in response to domestic violence risk to be associated with parent enablement. Customised care related to drugs and alcohol risk was not associated with parent satisfaction and enablement.

**Table 3:** PSQ and PEI by the provision of appropriate customised care in response to identified risks

**Customised care**	**PSQ (*n* = 261)**						**PEI (*n* = 265)**					
	n	mean	SD	median	IQR	P*	n	mean	SD	median	IQR	P*
***Smoking***												
Appropriate customised care	122	44.6	5.3	46.0	9	> 0.1	125	3.8	3.9	2.0	6.0	**0.032**
Inappropriate customised care	139	44.9	5.6	46.0	10		140	4.8	4.1	4.0	6.0	
***Mental health***												
Appropriate customised care	180	45.2	5.3	47.0	9	**0.027**	183	4.2	3.8	3.0	6.0	> 0.1
Inappropriate customised care	81	43.7	5.6	44.0	9		82	4.6	4.3	3.0	6.75	
***Domestic violence***												
Appropriate customised care	124	45.0	5.7	47.0	9	> 0.1	125	4.8	4.1	4.0	6.0	0.075
Inappropriate customised care	137	44.6	5.2	46.0	9		140	3.9	3.9	3.0	6.25	
***Drugs and alcohol***												
Appropriate customised care	149	44.8	5.1	46.0	9	> 0.1	151	4.2	3.9	4.0	6.5	> 0.1
Inappropriate customised care	112	44.8	5.9	46.5	10		114	4.5	4.2	3.0	7.0	

*IQR* Interquartile range, *SD* Standard Deviation

^*^Mann–Whitney U test performed

### Regression analyses

Ordinal logistic regression was performed to test associations between maternal mental health risk-related customised care and parent satisfaction, as well as between smoking and domestic violence risk-related customised care and parent enablement, whilst accounting for significant sociodemographic characteristics.

The results in Table 4 show that the total PSQ score was positively associated with mental health risk-related customised care. The strength of this association increased slightly after adjustment for the stratification confounders, geographic area and maternal parity, but decreased after the inclusion of selected sociodemographic fixed factors.

**Table 4:** Ordinal logistic regression models

Models	Fixed variables	Base model – adjusted^a^					Full model – adjusted^a^				
		Est	SE	Z	*P*	O.R. Exp(Est)	Est	SE	Z	*P*	O.R. Exp(Est)
**Total PSQ score**											
**1**	Customised care for mental health risk	0.54	0.24	2.26	**0.024**	1.72	0.42	0.24	1.77	0.077	1.53
	Parity	0.07	0.23	0.29	0.770	1.07	0.01	0.23	0.04	0.968	1.01
	Language						-0.76	0.44	-1.73	0.084	0.47
	No employment experience						-0.58	0.32	-1.83	0.067	0.56
**Total PEI score**											
**2**	Customised care for smoking risk	-0.44	0.22	-1.98	**0.048**	0.64	-0.39	0.22	-1.76	0.079	0.68
	Parity	0.27	0.23	1.18	0.237	1.31	0.28	0.22	1.25	0.211	1.32
	Language						0.66	0.40	1.65	0.098	1.93
**3**	Customised care for DV risk	0.41	0.22	1.85	0.064	1.51	0.43	0.22	1.94	**0.050**	1.53
	Parity	0.24	0.23	1.06	0.288	1.27	0.26	0.23	1.16	0.248	1.30
	Language						0.79	0.40	1.98	**0.048**	2.19

*Abbreviations: Est* Estimate, *SE* Standard Error, *O.R.* Odds Ratio, *DV* Domestic violence

^a^Models were adjustment for the stratification factors of geographic region and parity. Geographic region was included as a random variable, while parity, as a binary variable, has been included as a fixed factor

Language: language other than English spoken at home. No employment experience: mother had never had a job before

The univariate results show that both smoking risk-related customised care and domestic violence risk-related customised care are associated with the PEI score (Table 4). After adjusting for stratification confounders, the strength of the association with smoking care decreased slightly, while the strength of the association with domestic violence risk-related customised care increased. After the inclusion of the selected demographic fixed factor, domestic violence risk-related customised care was found to be significantly associated with increased parent enablement.

## Discussion

This study aimed to examine client outcomes as this related to parent satisfaction and enablement. It identified the relationship between sociodemographic characteristics and those outcomes, and the impact of customised care on those outcomes in the delivery of the sustained nurse home visiting program.

### Relationship between sociodemographic characteristics and parent satisfaction/enablement

The findings of this study indicated that language spoken at home (non-English) was a significant factor associated with lower parent satisfaction, but higher parent enablement. This result may be due to the issues pertaining to communication and the nurse-client relationship. Several studies suggest that the inability to understand the provider resulted in lower client satisfaction with the care received [46–48]. Language difficulties have also been shown to be associated with service avoidance, perpetuating poor health outcomes [49, 50]. A qualitative study [51] found that home visiting nurses reported difficulties in communication and in the building of relationships with culturally and linguistically diverse (CALD) mothers. Language barriers hindered the effective engagement of mothers with CALD backgrounds [52]. Improved knowledge of the unique needs and preferences of culturally diverse mothers, and the development of skills to work in ways that are curious, respectful and sensitive to difference, are vital to improving maternal satisfaction, and ultimately health outcomes for parents and children [53]. Moreover, Baker and colleagues [48] reported that the effective and timely utilisation of interpreter increased the client satisfaction. Thus, home visiting nurses require further support and mentoring to understand the diverse needs of families in relation to language and culture, and identify the additional service components and resources needed [54].

In contrast, mothers who spoke languages other than English at home had a higher sense of enablement as the result of participation in the program. Parent enablement is related to, but distinct from satisfaction [25]. The PSQ measures the extent to which expectations relating to the process of delivery of care have been met, rather than whether there has been any achievement of specific health outcomes. However, the PEI measures parents’ ability to understand and cope with their baby and their own health and life. Mothers who spoke languages other than English might have not been satisfied with the care process against their expectations, however, they might have felt their ability and confidence about their health and life had improved.

Mothers who had a job before had significantly higher parent satisfaction level. This may be because women with employment experience tend to have a better understanding of customer service in general, and thus their expectations of the services might be more realistic.

### Relationship between customised care and parent satisfaction/enablement

The results indicated that customised care was significantly associated with parent satisfaction and enablement.

#### Customised care in response to mental health risk

Mental health was the risk factor where the largest proportion (69%) of mothers received appropriate customised care, compared to 47% for smoking, 48% for domestic violence, and 57% for drugs and alcohol. Customised care in response to mental health risk was significantly associated with higher satisfaction. This may be because the risk identification or disclosure were accurate and customised care in response to the risk were appropriately provided by the nurses. Mental health risk is relatively more common [55] and nurses are better trained to support mental health needs [56, 57], and to enhance maternal mental health screening and referrals [58], compared to other risks such as domestic violence and drugs issues. Identifying mental health risk and customising the care in a more personalised approach in response to mother’s mental health status removes an element of stigma and thus might improve parent satisfaction.

On the other hand, the strength of the association between mental health-related customised care and parent satisfaction decreased after adjusting for confounders including language spoken at home (non-English) and no employment experience. This may be because women who spoke a language other than English and had no employment experience were correlated. Furthermore, these sociodemographic factors had a strong contribution to satisfaction with the care provided. Some studies have reported an increased prevalence of maternal mental health issues such as postpartum depression among CALD background mothers compared to non-CALD mothers [59–61]. In addition, no working in pregnancy is one of the risk factors for antenatal depression [62].

#### Customised care in response to domestic violence risk

Almost half of the mothers received appropriate customised care for domestic violence risk. There was a tendency for customised care in response to domestic violence to be associated with greater enablement. Furthermore, the regression analysis results revealed that customised care in response to domestic violence significantly increased the level of parent enablement when adjusted for confounding factors, which were language spoken at home (non-English) and no employment experience. The increase seen in significance when adjustment was made for language factor is interesting, as it supports the notion that women who spoke a language other than English can benefit from this type of intervention increasing their sense of enablement, particularly when given appropriately customised support in response to domestic violence risk. One in four migrant women with non-English speaking background experienced domestic violence in the first 12 months postpartum compared to one in six non-migrant women [59]. However, support services related to domestic violence do not cater well for the migrant and CALD background women [63]. As home visiting care is normally provided at home under a well-established and therapeutic nurse-client relationship, there might be more chances for nurses to observe or know of mother’s risk related to domestic violence. Thus, home visiting nurses hold significant opportunity to support in preventing mothers from adverse experiences related to domestic violence. It is therefore essential for nurses to gain better skills of culturally sensitive practice in relation to meeting the needs of women from diverse cultural, linguistic and religious backgrounds [63]. Furthermore, system issues such as lack of adequate referral pathways and limited access to supervision need to be addressed to support the delivery of effective intervention in response to domestic violence, particularly for non-English speaking women [54].

#### Customised care in response to smoking risk

Nearly half of the mothers received appropriate customised care for smoking risk. In contrast to the results related to the mental health and domestic violence risks, smoking risk-related customised care was significantly associated with lower parent enablement. There might be a few reasons behind. First, this result may be related to the customised care measure and categorisation. Smoking risk was identified based on maternal smoking status, but not partner’s or family’s smoking status. It is possible some nurses provided smoking related intervention based on the information or observation of other family member’s smoking status. Also, smoking intervention might have been provided intentionally even with mothers without smoking risk. This is because home visiting nurses might have considered that smoking prevention is critically important for all mothers, and it is also related to Sudden Infant Death Syndrome (SIDS), which is the third leading cause of infant death in Australia [64]. Second, indirect smoking is sometimes beyond mother’s control. Provision of the information that smoking is harmful to herself and her baby, but no ability to fully restrict the exposure to the smoking risk might result in lower enablement.

Moreover, the negative impact of smoking risk-related customised care on mothers’ enablement decreased in significance after adjustment for language spoken at home. This may be because the prevalence rate of smoking risk was lower among mothers who spoke a non-English language at home, who had higher level of enablement. Figures from the most recently published report from Australian Institute of Health and Welfare [65] state that people from CALD backgrounds (6%) are less likely to report daily smoking compared with the non-CALD population (12%).

#### Customised care in response to drugs and alcohol risk

Nearly 60% of mothers received appropriately customised care in response to drug and alcohol issues. We found no discernible impact of customised care in response to drug and alcohol risk on parent satisfaction and enablement. This may be, in part, due to ineffectiveness of the intervention in response to this risk. Prior research found that dealing with drug and alcohol issues is one of the most challenging interventions for care providers [66–68] and they have negative attitude about drug and alcohol issues [69]. Nurses are aware of information about definitions, signs and adverse outcomes of drug and alcohol risk, and available resources for individuals facing this risk through training sessions [70]. However, little training focused on strategies, approaches, or guidance on how to work with clients facing these issues [70]. Thus, the content related to the risk might have been provided, however, the quality of the intervention might not have met the needs of the mothers with the risk. Thus, there was no significant impact of the customisation on parent satisfaction and enablement.

### Implications for future practice and research

Our research suggests that it is important for home visiting nurses to provide precision care based on unique characteristics and risk factors in practice. Precision home visiting aims to customise evidence-based intervention programs for individual families [17]. This customisation is meant to improve the fit of a program for mothers’ and families’ unique needs, characteristics, and other key determinants of outcomes [71]. The sustained nurse home visiting service model needs to be flexible enough to cater for variations according to family circumstances and needs, particularly risk factors, while maintaining a core of evidence-based practice [72]. Based on the results from this study, customised care in response to identified risks and needs is recommended as essential to effective home visiting practice, together with fidelity to core components of the program. Thus, we suggest that nurses need to be supported to further develop their capacity regarding both identifying families’ risks and needs, and competence in providing timely intervention and referral for families in need of this form of support.

In addition, more knowledge about and evidence for precision home visiting is required. Precision home visiting can use research evidence to identify what works best, for whom, and in what contexts to achieve specific outcomes across diverse families [16, 17]. It focuses on the components of home visiting services that are most likely to be effective in light of mothers’ and families’ sociodemographic and cultural characteristics and context [18]. There is an emerging demand for new home-visiting strategies to address the diverse and critical needs of mothers and families to improve parent satisfaction and enablement. Thus, we recommend further research to investigate the specific care components which are required to address mothers’ and families’ critical needs for ensuring effective outcomes of the program. For example, future research may seek to identify the customised components and schedule, which are effective for groups of mothers with specific risk factors such as CALD backgrounds, mental health issues, and risk for domestic violence, in addition to core ingredients. Furthermore, there is a need to explore the home visiting nurse’s decision-making process related to the customisation of care.

### Limitations and strengths

The findings of this study need to be interpreted in light of its limitations. First, these findings are considered within the context of the home visiting program, the right@home, which was conducted in two states in Australia. In addition, we included the data of the participants who had completed the full intervention until child age 24 months. Therefore, findings may be subject to selection bias. Second, the risk factors were identified based on the screening data and nurse’s records of a risk disclosure. There might be mothers who were reluctant to disclose their risks. Thus, there might be undisclosed or unidentified risks. Meanwhile, this study considered provision of customised care if at least one episode of care was provided in response to each maternal risks during home visits. We did not consider the frequency or amount of content provided in response to identified risks and focused on whether or not the content was provided when the maternal risk was identified. Third, this study identified several factors related to parent satisfaction and enablement, such as language, employment experience, and customised risk-related care. However, there may be other factors, which were not measured in this study, that improve client outcomes, for example, effective relationship between mothers and care providers, and providers’ characteristics. Fourth, this study used the content data recorded by home visiting nurses and did not compare to direct or objectively observed practice during home visits. Therefore, the content may be biased by the nurse-reported nature of the data. Previous research under the Mother and Infant Home Visiting Program Evaluation (MIHOPE) explored various measures of home visiting content and process and found that videotaped records of home visits allowed an independent and reliable observation of the content and process of home visiting [73–75]. Therefore, future research may seek to validate the reliability of the tools via random direct observations of home visits by clinical supervisors, observations from video recordings, and/or mother’s feedback on care experience during home visits. Lastly, this study explored “customised care” using the existing data and information with mothers categorised into two groups whether or not they received appropriately customised care based on the identified risks and provision of care. We acknowledge that there may be a difference between groups A and D as well as B and C in Fig. 1. Specifically, our study did not determine whether the group A condition of customised care (care associated with risk) was the same as the group D condition (non-care associated with no risk). Rather this study was scoped around assessing whether getting service relating to mother’s needs or not improved satisfaction and enablement. We suggest future studies should further explore quantitatively and qualitatively whether mothers and families experience differently the meeting of their needs or absence of needs.

Nevertheless, this research considers aspects of the home visiting process which are not well conceptualised in previous literature by exploring the association of customisation with satisfaction and enablement using an observational design. The findings on the relationship between customised care in response to identified risk factors and outcomes contribute to accumulating the knowledge and evidence of precision care.

## Conclusion

This study contributes to advancing precision home visiting research by adding new evidence to the existing body of empirical research that examines the relationship between the care processes and client outcomes, such as parent satisfaction and enablement in the delivery of home visiting care. Our results showed that appropriately customised care in response to client risks and needs improved the level of parent satisfaction and enablement. Quality of care can be achieved by providing the core components of care, in a way that is appropriately customised based on client risks and needs. Together with ensuring the fidelity to the core program components, the flexibility to modify the care based on diverse risks needs to be allowed in the design of sustained home visiting programs to provide better quality of home visiting care.

## Data Availability

Data cannot be shared publicly because of ethics and program licencing requirements to maintain the confidentiality of participants and participating health services. However, data are available from the Murdoch Children’s Research Institute Institutional Ethics Committee (contact via Dr. Susan Perlen at susan.perlen@mcri.edu.au) for researchers who meet the criteria for access to confidential data.

## Abbreviations

IQR: Interquartile range
MECSH: Maternal Early Childhood Sustained Home-visiting program
MIHOPE: Mother and Infant Home-visiting Program Evaluation
RCT: Randomised Controlled Trial
PSQ: Patient Satisfaction Questionnaire
PEI: Patient Enablement Index
REML: Restricted Maximum Likelihood
CALD: Culturally and Linguistically Diverse
SIDS: Sudden Infant Death Syndrome
